# Three-Dimensional Reconstruction of Individual Particles in Dense Dust Clouds: Benchmarking Camera Orientations and Reconstruction Algorithms

**DOI:** 10.3390/jimaging5020028

**Published:** 2019-02-13

**Authors:** Michael Himpel, André Melzer

**Affiliations:** Institute of Physics, University Greifswald, 17489 Greifswald, Germany

**Keywords:** stereoscopy, particle tracking, dusty plasmas, complex plasmas, multiview geometry, 3D, computer vision, plasma diagnostic

## Abstract

In dusty plasmas, determining the three-dimensional particle positions and trajectories of individual particles is often required. This paper benchmarks two approaches capable of reconstructing the trajectories of particles in three dimensions. The influences of the particle number, the particle number density, and the orientation of the individual cameras are studied. Additionally, the demands on the desired image quality, required for these algorithms, are discussed. The reader is given practical information for the appropriate reconstruction approach and camera positioning that should/could be used in a specific application.

## 1. Introduction

When microparticles are suspended in a plasma (partially ionized gas), one usually speaks of a dusty plasma. Such microparticles attain high charges and, thus, interact electrostatically. Imaging of particles in a dusty plasma has been an important subject, ever since the first observations of microparticle structures in a laboratory plasma [1,2,3]. While the studied phenomena might have changed in recent decades, the basic task has not: The position and motion of the dust particles in a plasma have to be determined as accurately as possible, since the particle motion reveals the underlying forces and interactions [4,5]. As the particles are typically micrometer-sized and separated by some hundred micrometers, a dusty plasma that occupies several cubic centimeters is optically thin. This allows viewing of individual dust particles [6,7,8]. Nevertheless, from a single viewing direction, mutual occlusion of particles is a frequent phenomenon. Hence, it is reasonable to apply multiple camera views (“stereoscopy”) to reconstruct the 3D positions of particles in a dust cloud. The cameras are usually tens of centimeters away from the observed volume, as the experiment is done in a vacuum chamber.

In recent years, two main approaches have been used to determine particle trajectories in many-particle structures: Triangulation and, more recently, iterative reconstruction [9,10,11]. In this paper, both approaches will be benchmarked in order to help other experimenters in choosing and building appropriate setups for their experiments. Questions of interest are: Which algorithm works best under specific conditions, and how should the cameras for observing the 3D dust cloud be arranged. These questions will be addressed in this paper.

The software that was used for the multiset and the iterative reconstruction is written in MATLAB computing language, and is freely available for download [12].

## 2. Algorithms

To reconstruct the three-dimensional position of a particle from its two-dimensional projections, one needs at least two different views. In this paper, we only consider measurements with three or four views, as this is known [13] to be more robust with many visible particles in the camera images. Furthermore, we imply that the camera calibrations are known. These calibrations are a set of parameters that are usually experimentally determined by viewing a specific calibration target [14,15].

### 2.1. Triangulation-Based Algorithm

The triangulation starts from the two-dimensional particle positions in the camera images. For the determination of particle positions, sophisticated procedures exist, which are tolerant of image noise [16,17,18]. In the next step, the basic idea behind the triangulation-based algorithms is that a particle is seen along different line-of-sights when viewed by two cameras at different angles. The 3D position is then found from the intersection of these lines-of-sight. The triangulation from the views of the cameras is basically a triangulation from two views [19], which combines the information from all camera pairs {1 + 2, 2 + 3, 1 + 3}. The final triangulated position is then the mean of the three triangulated positions from each subset. The main point of using three cameras is, however, not a better resolution of the particle position. Three cameras are required for a unique identification of particle correspondences. As all particles appear similar in the camera images, it is neccessary to establish methods to identify which particle, seen in camera $C_{2}$, is the image of the particle seen in camera $C_{1}$. This problem can only be solved with the help of a third or fourth camera when many particles are imaged [13].

It has been found that one can considerably improve the reconstruction of dense particle structures by using at least four cameras with a so-called “multiset-triangulation” [13]. The computationally most effective approach is not to calculate the triangulation from the four views directly, but to generate 3-camera subsets {1 + 2 + 3, 2 + 3 + 4, 3 + 4 + 1, 4 + 1 + 2, 1 + 3 + 4, 2 + 4 + 1}, and to perform the above mentioned reconstruction from three views for each of these six camera subsets. A particle is then considered to be recovered when it appears in at least a certain number, typically four out of six, of the subset reconstructions. This algorithm is found to generate accurate positions, even in relatively dense particle structures with many particles [20] and particle occlusion. In the benchmark tests, shown here, we will exploit the information from three cameras on one hand, and from four cameras on the other hand.

The last processing step (namely, particle tracking) is required in both situations, and denotes the process of linking particle positions in consecutive frames to generate trajectories. Different linking algorithms, such as nearest-neighbor or Wiener-filtering [21], are possible. Nearest neighbor tracking is only considerable when the motion from one frame to the next is much smaller than the interparticle distances, which can generally not be assumed. The Wiener-filter basically smoothes the position data of the particles and then extrapolates a consecutive position for the next frame. Even better tracking performance can be achieved when a statement regarding the acting forces can be implemented in the position prediction. This can be done using a Kalman filter [22]. It predicts the next particle position for a trajectory in 3D using a given model (constant acceleration or constant velocity), followed by the correction of the model with the measured particle position. Then, from the trajectories one can obtain the velocities and accelerations of the particles and, hence, the underlying forces can be determined. Moreover, the linking process can significantly reduce erroneously reconstructed particles—called “ghost”-particles, because such particles typically appear only in very few consecutive frames. When rejecting particle trajectories that exist only for short period, one will remove these erroneous “ghost” detections. However, one may also discard some true short-length trajectories. The influence of the minimum trajectory length on the reconstruction quality will be also discussed.

### 2.2. Iterative Particle Reconstruction

Iterative particle reconstruction algorithms work quite differently, compared to the triangulation-based method. Here, the full information of the particle’s projection in each of the camera images is used, rather than reducing its information to the position.

For the application in the field of dusty plasmas, we adopted the so-called “shake-the-box”-algorithm, which is based on iterative particle reconstruction. Detailed information regarding this algorithm can be found in more specific publications [10,11,23]. Here, we will only give a very brief description of how the algorithm works.

To start with the analysis, one needs a prediction of the three-dimensional particle positions. This is usually done in practice using multiset triangulation analysis, as described in the previous section. In the situation described here, the initialization with multiset triangulation was performed for the first 5 frames, then the iterative procedure sets in. Based on a Kalman-filter approach, a position prediction is made for the next frame. This predicted position is then used to generate an artificial image where the particles are modelled as a Gaussian distributed pixel intensity at the particle location. In a small evaluation window, somewhat larger size than the particle projection, the modelled image is then compared to the measured image for each particle individually. This comparision is then used to optimize the particle position.

By optimizing (“shaking”) the 3D-position of every single modelled particle, the deviation between the measured and the modelled image is minimized. Note that this iterative prediction-correction workflow can be regarded as an implicit tracking procedure, comparable with the triangulation approach. To account for particles that have recentlyly entered the observation volume or that have not been tracked yet, multiset triangulation is applied to a residual image. The residual image is obtained by subtracting the modelled image of all yet optimized particles from the recorded image. When new 3D particle positions can be found using multiset-triangulation in the residual image set, those start a new trajectory.

This algorithm is very potent, especially for high particle numbers. The main computational load is generated by computing the particle images and comparing them to the measured images. These tasks can be easily parallelized, nevertheless it is possible to handle the reconstruction of several thousands of particles on an office PC in reasonable time.

## 3. Camera Setups

**Figure 1:**
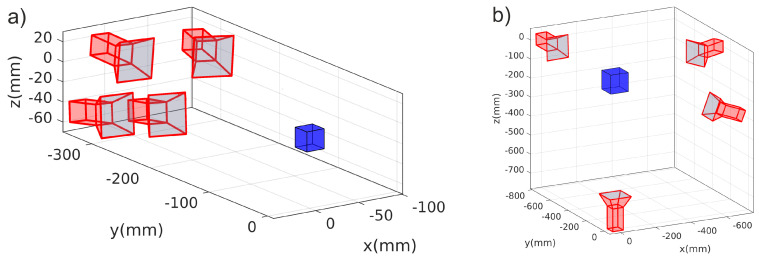
Two different possibilities for orienting four cameras. In (**a**), a narrow-angled setup is shown. This setup can be used in applications where only a single optical access to the investigated object is possible. In (**b**), the setup is wide-angled instead. This positioning can be used when different optical paths allow investigation of an object from very different angles. The blue box visualizes the observation volume, which is enclosed in a vacuum chamber in laboratory experiments.

When three or more cameras are used for stereoscopic investigations, it is often simplest to mount the cameras on a common rig with slightly varied viewing angles. Such a narrow-angled setup is shown in Figure 1a. This is a four-camera setup that we use in parabolic flights where the cameras have to share the view through one window of the plasma chamber. As in many other experiments, this approach keeps the other diagnostic ports available for different diagnostics. A drawback of this camera placement is that one expects a larger reconstruction inaccuracy along the viewing direction, due to the narrow viewing angles.

In the wide-angled camera setup, the individual cameras are positioned in such a way that they observe the common volume from very different angles. Such a wide-angled camera setup, with four cameras, is shown in Figure 1b. This four-camera setup is used in our laboratory experiments. Of course, this setup requires optical access to the dust cloud from many directions (thus blocking access for other diagnostics). In total, three windows are occupied by this camera setup. Due to the different viewing angles, the error of the reconstructed particle positions is expected to be isotropic and relatively small.

The cameras that we use in our experiments, as well as in the simulations, have a 1280×1024px-CMOS sensor. For the sake of aquisition speed, we only use 8bit-intensity information from the sensor. The lens setup is chosen to generate a spatial resolution of about 10μm per sensor pixel.

## 4. Benchmark Scenario

To evaluate the capabilities of the reconstruction algorithms under the different viewing geometries or parameters, the following benchmark situation is provided.

The processed dataset contains a cloud of 21,122 particles, placed on a face-centered cubic (fcc) grid. The mean particle distance is 500μm. After this initial positioning of the particles, random noise is added to the particle positions from frame to frame. This is done for 100 frames. The resulting trajectory can be interpreted as particles that are subject to Brownian motion [24,25]. This crystalline particle structure covers a spherical volume with 10mm diameter. This volume is chosen large enough to cover the full field-of-view for both camera arrangements.

**Figure 2:**
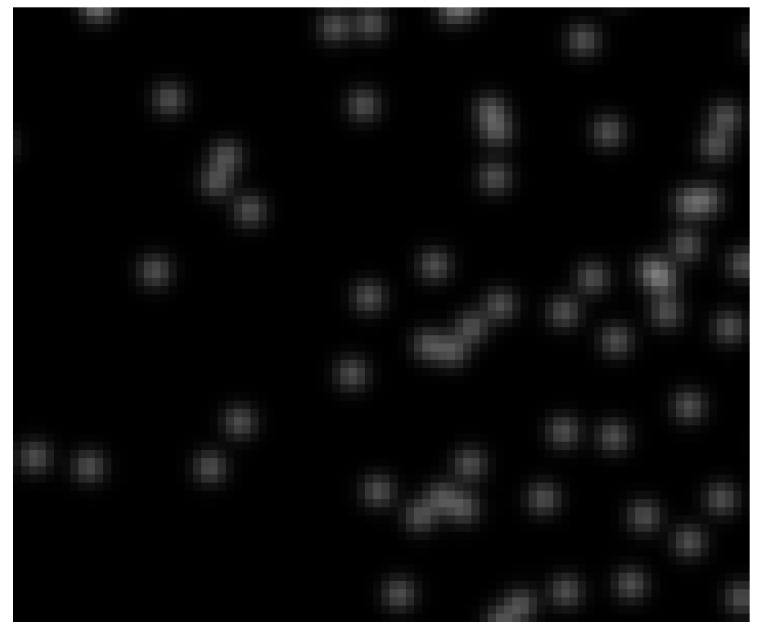
Artificially generated measurement image (a fraction of 75×90px of the full image with 1024×1280px). The intensity of each particle is Gaussian distributed around its projected position.

To obtain significant benchmark tests that can be compared to real measurements, the analysis has to be done with modelled images that are comparable to the ones obtained in experiments. The artificial images are generated in the following way: Having the camera calibrations [15], it is possible to project the three-dimensional positions of all particles in a certain frame onto the camera image. Using these exact positions, an image is generated, where each particle is modelled with a Gaussian distributed pixel intensity in an evaluation window of 4×4px. The width of the Gaussian is 4px and the peak intensity is 30% of the pixel saturation. These parameters are chosen to obtain particle images similar to those typically generated by imaging dust particles, with a size of 3–10 μm. An exemplary image is shown in Figure 2.

In every benchmark run, we will present three quantities to describe the quality of the reconstruction algorithms. The first quantity is the position error. Here, the error denotes the root-mean-square displacement of the reconstructed particle position, compared to the true particle position which is known from the input data used to generate the artificial images. Please note that the artificial images have been created without background noise. This was made in order to be as independent as possible from the imaging hardware, which is the main origin of noise in the images. The drawback is, of course, that the accuracy seems quantitatively better in our benchmarks than will be possible using real measurements. A reconstruction from real measurement data is discussed in Section 6.

The second important quantity is the percentage of recovered particles. A particle is defined to be successfully reconstructed when the position deviation is less than 5μm, otherwise it is considered to be a ghost particle. Then, the number of successfully reconstructed particles $N_{r}$ divided by the total number of visible particles in the data set $N_{\mathrm{data}}$ is defined as the percentage of recovered particles, $N_{r}/N_{\mathrm{data}}$.

The last presented quantity is the percentage of occuring ghost particles. This is defined as the number of unsuccessful reconstructions $N_{g}$ divided by the total number of reconstructed particles $N_{g}/N_{r}$. Please note that, when comparing the position error or the ghost particle rate, it must be seen in relation to the total number of reconstructed particles. If, for example, only very few detections are reconstructed, their positions might be more accurate because only optimal particle images have been accounted for.

In this paper, we will use absolute units for the reconstructed particle positions, the particle density, and the position error. These absolute units generally reflect the situation of 3D clouds of micron-sized dust particles in a plasma discharge. To scale our results to a drastically different imaging setup, it may be more suitable to convert our findings from metres to sensor-pixels. In the cases discussed here, one pixel corresponds to an absolute value of 10μm.

## 5. Benchmark Results

Here, we show the results of the benchmark runs for different situations. The runs were performed using the full multiset triangulation with four cameras (“multiset”), the simpler triangulation using only three of the four available cameras (“3cam”) and the shake-the-box algorithm (“STB”). The computation time was very different for the algorithmsm and depended on the particle number and density. In general, one can say that the STB algorithm computation time scaled with *N* and the triangulation based methods scaled with $N^{2}$. The multiset approach was based on six permutations of a 3cam-triangulation, which resulted in a rough factor of 6 in the computation time, compared with the simpler 3-cam approach. For a 2000-particle cluster with intermediate density, the processing time of our code ranged from ≈10min (STB) to 1–10h (3cam, multiset).

Only trajectories with a length of 20frames or more were considered in the following benchmarks runs.

### 5.1. Particle Number Density

**Figure 3:**
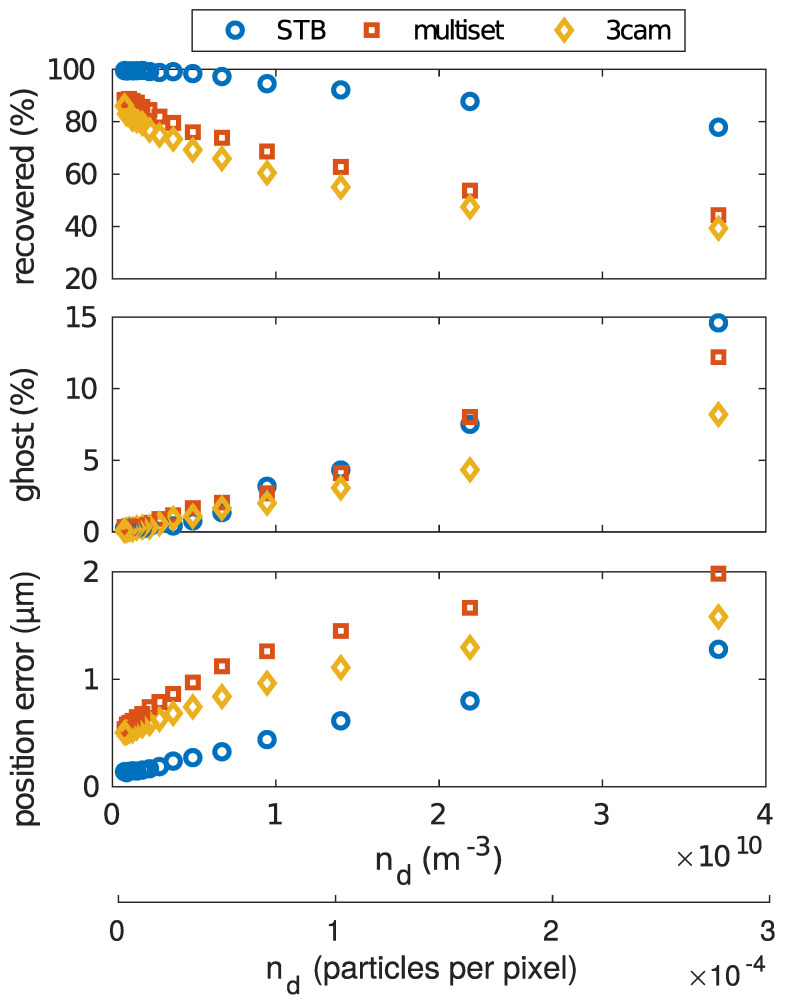
Comparision of the different reconstruction algorithms. While the particle number density $n_{d}$ was varied, the position error, the percentage of the reconstructed particles, and the percentage of ghost particles were determined. The particle number was 500 for all data points.

We start with a situation where the apparent dust density in the camera images was varied. This was realized by picking a constant number of 500 particles from the dataset and scaling their 3D-coordinates. The generation of the artificial particle projections in the images was always done using the same parameters, so the apparent size of the particles in the images was unchanged. The result of the particle coordinate scaling was a dust cluster that filled a larger or smaller volume, depending on the scaling factor. Thus, the 2D particle density (“seeding density”) of the particles in the camera images increased or decreased. Along with this, the image area filled by the 500 particle dust cloud changed. This benchmark is especially interesting for the triangulation-based approaches. There, the particle density in the 2D images, given usually in particles per pixel, was an important limiting factor. If the seeding density got too high, it was more and more difficult to identify particle correspondences. This is shown in Figure 3, by the fast decrease of the recovered particle number at increased particle number density. Here, the multiset and 3cam algorithms showed a much faster decrease of successful reconstruction, compared to STB.

Further, all algorithms were subject to a similar increase of ghost particle detections. Finally, the positioning accuracy was slightly higher using the STB algorithm, but it was generally at a very high level, as the position error was less than 2μm in all cases.

**Figure 4:**
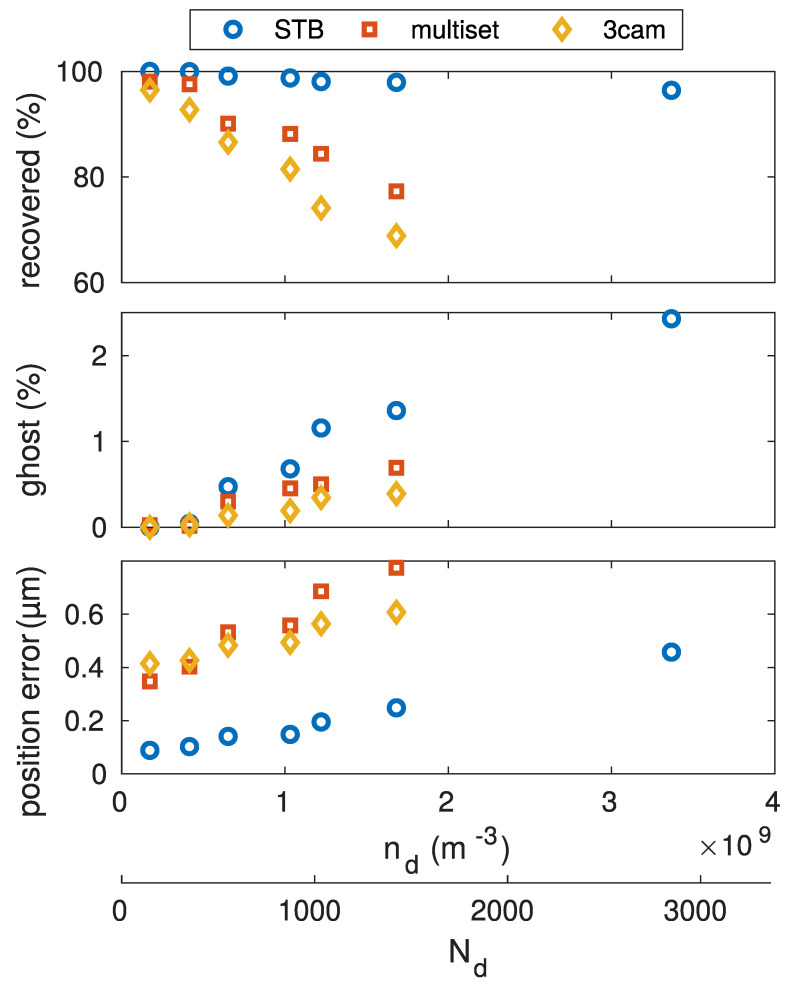
Comparision of the different reconstruction algorithms. While the particle number density $n_{d}$ was varied, the positioning accuracy, the percentage of the reconstructed particles, and the percentage of reconstructed ghost particles were determined. The crystalline dataset with a wide-angled camera setup has been used as basis data.

The second benchmark was carried out to show the performance of the different algorithms regarding the variation of the particle number density in a static volume. The particle density was much lower than in the previous case, but the total number of particles to be reconstructed became larger. Here, the volume filled with particles was kept constant and the total particle number was varied. This situation can be found experimentally when a given volume is illuminated and observed, but the density of the dust structure can be altered (i.e., by using different particle sizes). Figure 4 shows the results that were achieved with the different algorithms.

The STB algorithm reached a stable high level of reconstruction accuracy and number of correctly recovered particles. The number of ghost particles was also kept to a relatively low level, at about 2%. The triangulation-based algorithms showed a clear decrease of correctly recovered particles as the number density increased. The accuracy of the reconstructed positions was also very good and stayed below 1μm in deviation. As all trajectories with a length less than 20 frames were dropped, the amount of falsely identified particles (ghosts) was kept below 1%.

### 5.2. Absolute Particle Number

**Figure 5:**
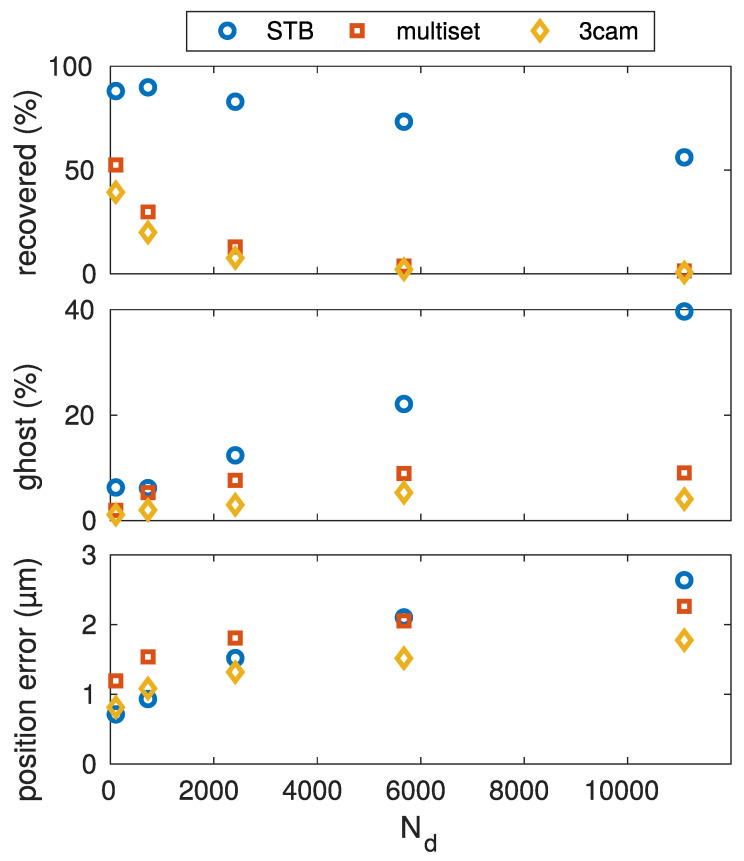
Variation of the total particle number, while keeping the number density constant. The constant number density is comparable to that of a microgravoty dust-cloud.

Now, the absolute particle number was varied, keeping the number density constant at a quite high density of $n_{d}=1\times 10^{9}\;m^{-3}$. For this, the full dataset was cropped, so that only a portion, with varied radius, of the full dataset volume was considered. The constant density was comparable to what is typical for a dust cloud under microgravity conditions, filled with micrometer-sized particles. The resulting benchmark test is shown in Figure 5. The low reconstruction yield for the multiset and 3cam algorithms, even at low particle numbers, was a result of the high number density. It can be seen that the STB algorithm was significantly better at reconstructing particles in this situation. The reconstruction number decrease with increased particle number was almost linear for STB, but stronger for the triangulation based methods. The amount of reconstructed ghost particles, however, was larger with the STB algorithm and reached a stable level with the triangulation approaches. The position error was small for all three algorithms.

### 5.3. Influence of Camera Setup

**Figure 6:**
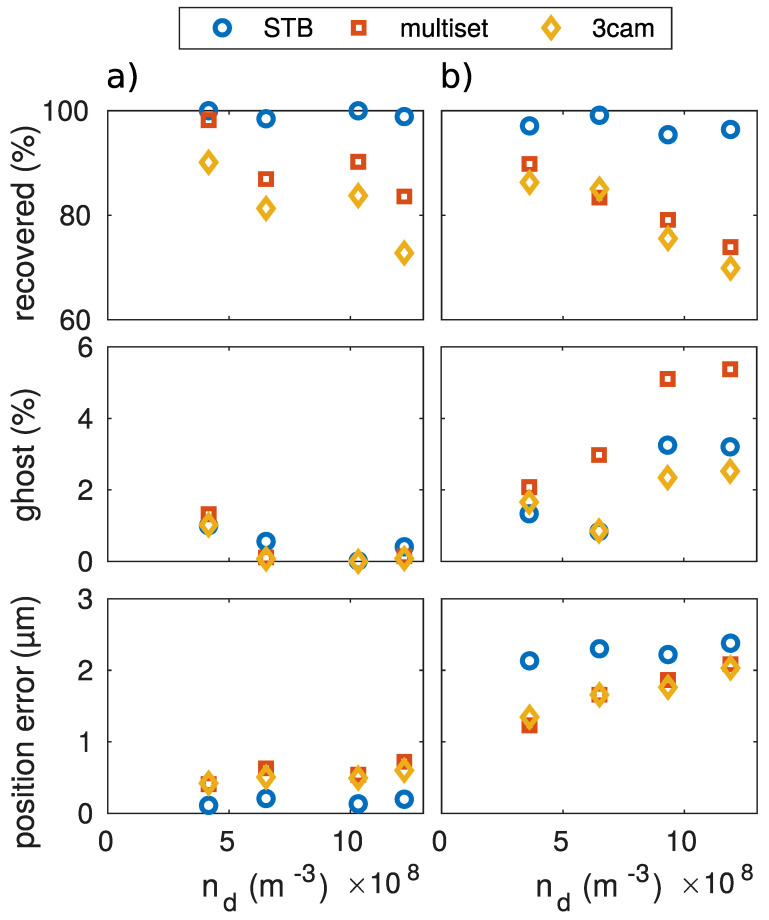
Comparision of different camera orientations. The results obtained with the wide-angled camera setup are shown in column (**a**) and the results from the narrow-angled setup are shown in column (**b**).

In this section, the influence of the camera orientation was tested for particle numbers of 250, 500, 750, and 1000. In Figure 6, all reconstruction methods showed a smaller position error when the cameras were positioned at wide angles to each other (Figure 6a). The narrow-angle camera setup (Figure 6b) showed a significantly higher position error. The narrow-angled camera setup also showed a higher number of ghost particles, compared to the wide angled-setup. The initial hypothesis, that cameras with very different viewing angles should have a higher accuracy, was thus confirmed.

### 5.4. Trajectory Length and Ghost Particles

**Figure 7:**
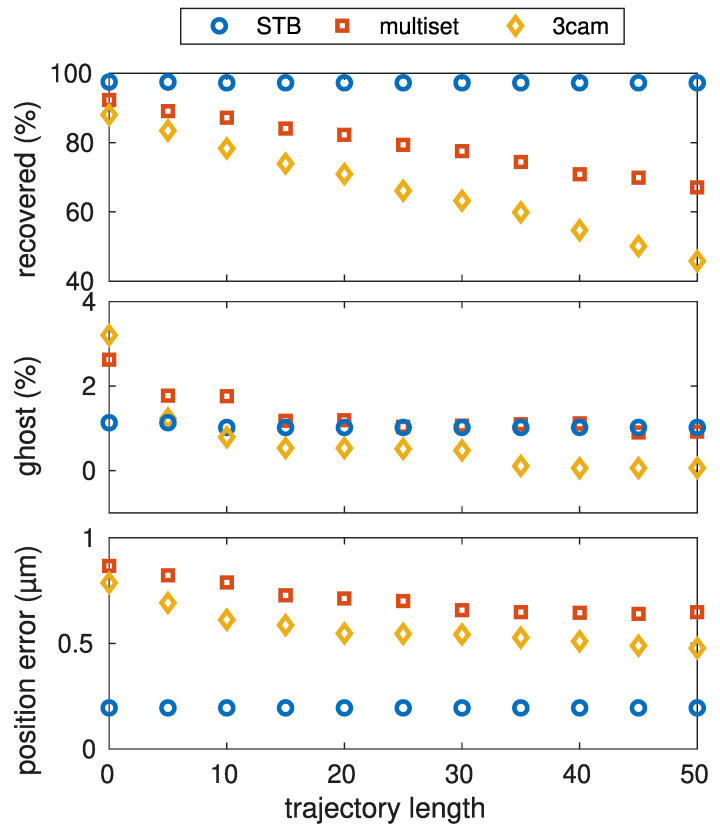
Influence of the minimum trajectory-length criteria on the reconstruction result for a 1000-particle cluster. The shake-the-box algorithm is quite unaffected. The single particle tracking algorithms show a negligible amount of ghost particles when the minimum frame length is above 20frames. Unfortunately, the percentage of recovered particles also declines when the minimum frame length is increased.

The result of each reconstruction algorithm is a set of particle trajectories. Generally, it is not possible to determine whether a trajectory reflects the motion of a real particle or is built from erroneous detections. As an additional constraint, one can try to consider trajectories only as real when they are tracked for a certain minimum length, as erroneous detections only occur in a limited number of consecutive frames. To estimate the influence of this minimum length condition, Figure 7 shows reconstructions of a 1000-particle structure. The total length of the processed data is 100 frames. For each algorithm, trajectories with a minimum trajectory length of 0–50frames have been excluded from the dataset.

The trajectory sets resulting from the triangulation-based reconstruction approaches show a strong influence. When short trajectories are excluded, Figure 7 shows that the occurence of ghost particles can be significantly reduced from 2–3% when selecting all trajectories as valid, to below 0.5% when all trajectories shorter than 20 frames are discarded. As ghost particles result from erroneous detections, the position error decreases when ghost particles are excluded from the trajectory set. Unfortunately, correctly determined particle positions also get excluded this way. Figure 7 shows a strong decrease of the recovered particle percentage with increasing minimum trajectory length, indicating that more and more correct reconstructions are excluded. The best choice for the minimum trajectory length is a trade-off between a high number of recovered particles and a small number of ghost particles.

In contrast, the “shake-the-box”-algorithm results in very long trajectories for the majority of the particles. Hence, this trajectory set is quite unaffected by choosing a particular minimum length condition, as the resulting trajetories are very long and mostly cover the full 100frames. When the trajectories from the STB algorithm are fragmented, due to more difficult reconstruction conditions, the ghost particle percentage, as well as the position error and the recovered particle number, will decrease with an increased minimum trajectory length (comparable to the triangulation algorithms).

### 5.5. Influence of Image Noise

**Figure 8:**
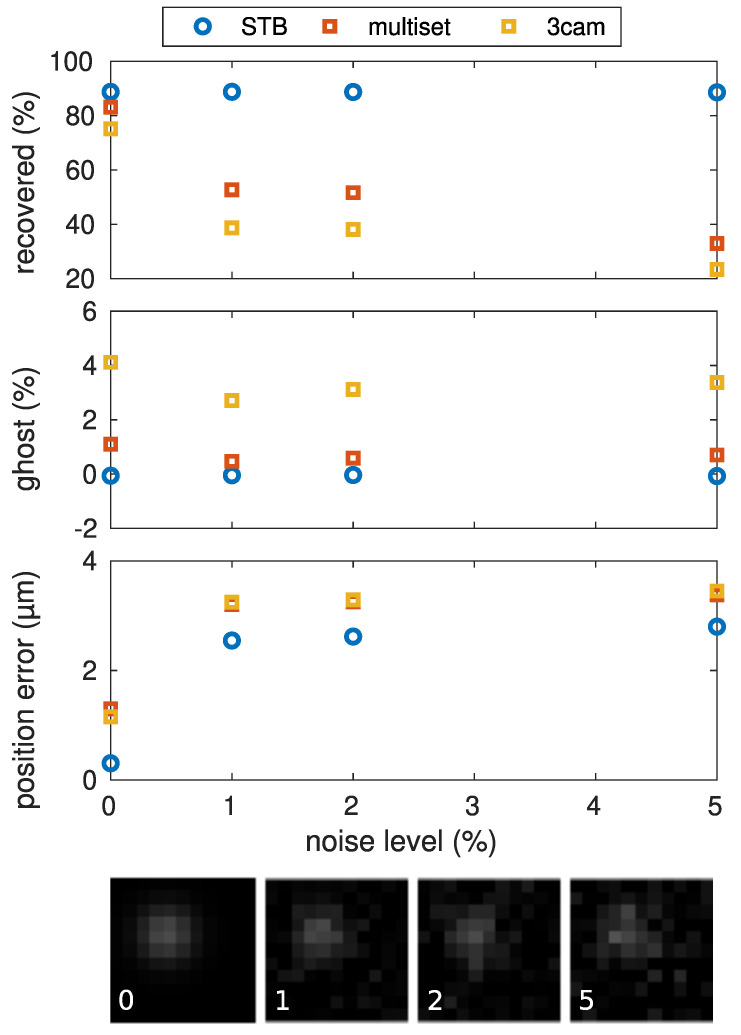
Influence of Gaussian image noise on the algorithms. Below the graphs, the sample images show the particle appearance with noise levels 0%, 1%, 2%, and 5%.

This benchmark run was done to estimate the performance of the algorithm in the presence of background noise in the recorded images. For this, Gaussian noise with zero mean and varied variance $\sigma^{2}$ was added to the artificially generated images before all other processing steps. Figure 8 shows the reconstruction performance for a 1000-particle system, depending on the noise level $\sigma^{2}$. As in the other benchmark runs, trajectories shorter than 20 frames were discarded. This had a large impact on the multiset reconstruction in the presence of noise, as missing detections (due to noise) in only a few frames led to the discontinuation of trajectories. Thus, many short but possibly true trajectories were excluded by the trajectory length condition in this benchmark run. This is clearly seen in the decrease of number of particles that were recovered with the multiset algorithm. In contrast, the number of recovered particles obtained by the STB algorithm was unaffected, even with higher noise levels. The amount of ghost particles was small for both algorithms. The position error shows that the multiset algorithm was relatively unaffected against noise and can be compared with the STB algorithm, mostly. The 3cam algorithm performed very similarly to the multiset algorithm, except for the overall lower reconstruction percentage. The multiset and 3cam algorithms had the same position error. This was due to the fact that both approaches used the two-dimensional particle positions as a common data basis, which was distorted by an increased influence of the noise.

## 6. Experimental Results

**Figure 9:**
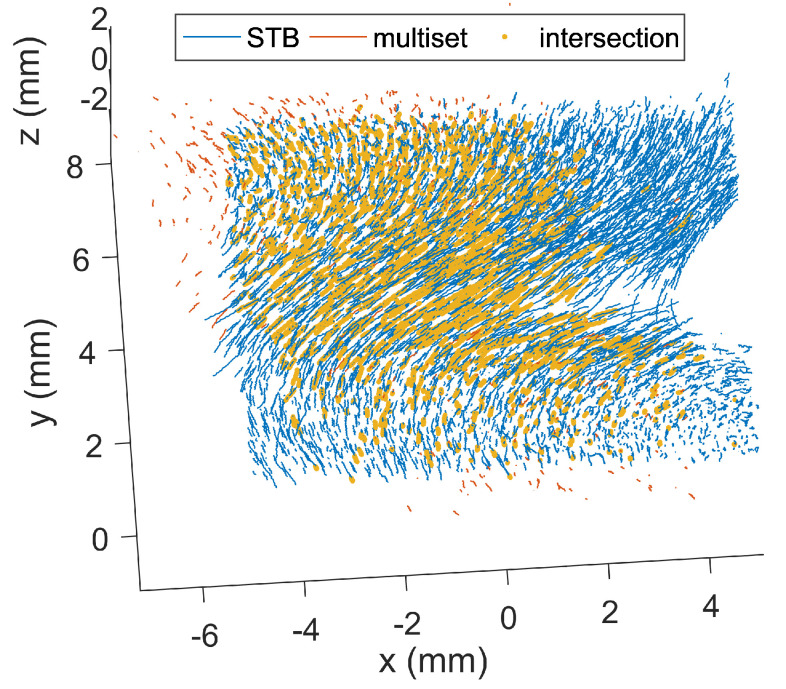
Trajectories obtained from STB (blue) and multiset (red) reconstruction algorithms. The particle positions reconstructed by both algorithms are emphasized by yellow markers.

To give an example of how the different algorithms performed with real data, they have also been applied to measurements from a dust cloud investigated under microgravity conditions [23]. The images were taken with a narrow-angled setup, and show a 14×8×2mm illuminated section of the larger dust cloud. The particles were observed through a single window in the plasma chamber. An image analysis indicates that there were approximately 5000–6000 particles visible in each camera image. Thus, the resulting particle number density was about $2\times 10^{10}\;m^{-3}$. The maximum detectable number of particles was somewhat smaller, as the cameras did not share exactly the full field-of-view, for geometric reasons. A total number of 100 frames were investigated, as in the benchmark section. In the experiment, the particles had a net drift and, thus, left the measurement volume after a short time. Hence, the trajectory length filter excluded a lot of trajectories—as a result, the amount of reconstructed trajectories was smaller than the maximum possible trajectories. The shake-the-box algorithm reconstructed about 2000 particles in every frame, and the multiset algorithm reconstructed 500 particles. The resulting trajectories are shown in Figure 9. As there was no true particle position available to compare the data with, both measurements can only be compared with each other. The STB algorithm recovered 75% of the particles that were found with the multiset algorithm, while 80% of the STB-particles had no counterparts in the multiset data. The other way around, only 17% of the STB data was recovered by the multiset data and 33% of the multiset data had no counterparts in the STB data. Figure 9 suggests that nearly all multiset trajectories that were in the actual illuminated dust cloud volume were found by the STB algorithm. The extra trajectories obtained by the multiset algorithm seem to have been established by wrong particle correspondences and, thus, lie even outside the illuminated dust cloud region. The position error of the two approaches can only compared against each other. The mean distance of a particle reconstructed by STB and its corresponting partner in the multiset algorithm was about 14μm. This error is larger than the values obtained from the artificial benchmarks, as the real measurements include image noise which led to deviations in the 2D positions. Additionally, minor errors in the camera calibrations increased the position error in the real measurements.

The computation took about 15 min with the STB algorithm and about 5 h with the multiset algorithm, on a regular office PC. The overall performance of the STB algorithm can, thus, be declared superior when compared with the multiset algorithm.

## 7. Conclusions

The presented algorithms processed camera images, with particles appearing as blobs in the camera images having a higher intensity than the background. There are many influences that may result in imperfect imaging. Table 1 shows a summary of the typical deficits, and indicates whichever of the algorithms are applicable.

The iterative reconstruction algorithm was most demanding, regarding the image quality. Generally, the particle shape must be suitable to be reconstructed by a Gaussian intensity profile. This constraint implies two basic demands: First, the particle image must be disc-shaped. Second, the highest intensity must be located in the center of the disc. This condition is not met for unfocused particles, which are projected as a ring rather than a disc. In this case, the application of the iterative approach is not recommended.

The particle intensity on the sensor is another parameter that can be chosen by the experimenter. The recommended intensity of a particle is about only 30% of the saturation intensity of the pixel. As the intensity of the scattered light adds up on a pixel, with a maximum of 30% pixel intensity it is possible to collect the light of three particles behind each other without saturating the sensor. In the iterative procedure, the modelled intensity distribution is subtracted from the image. Hence, these three particles in a row can be discriminated. When single particles generate saturated pixels on the sensor, particle occlusion can not be handled reliably. The particle intensity, however, must not be too small. Otherwise, it cannot be discriminated against the background. Additionally, the particle intensity should be relatively constant, which is experimentally realized by a power-stabilized laser as light source. The intensity is a particle-specific property, which is also tracked along the trajectories. Fast-changing intensities are interpreted as an erroneous particle detection. Such fast-changing intensities also occur when the particle only covers very few pixels. This happens when the magnification is not chosen properly. Image noise is tolerable, to a certain extent.

The triangulation-based algorithms were generally more tolerant, regarding difficult imaging conditions. The only processing step that has to be done with the actual measured images is the detection of the two-dimensional positions of each of the particles. Every further step relies on the two-dimensional particle positions in each camera view. Though the 2D-position detection is the most accurate when a particle appears as a Gaussian distributed pixel intensity distribution, a fairly accurate detection (0.5–1 px deviation) is possible, even with problematic particle projections. By applying a two-dimensional Gaussian filter, it is possible to obtain a reasonable position estimation even for defocused, saturated, and non disc-shaped particle images. However, the signal-to-noise ratio should be low enough so that the particles can be discriminated against the background. Usually, sophisticated detection algorithms [16] can assure this, even for relatively high SNR conditions.

Regarding the presented benchmarks, iterative reconstruction is clearly the preferable algorithm. However, it is not possible in all experimental conditions to generate images that have sufficient image quality for the iterative reconstruction. Under difficult imaging situations, one should use multiset triangulation instead. Especially with real measurement data, the triangulation from many sets of camera permutations can significantly improve the reconstruction results at high particle seeding rates, compared to the triangulation from only three cameras. When, for some reason, one can not apply the STB algorithm, the number density may be virtually lowered by using tracer particles (e.g., Rhodamine-B marked particles [26]). When ghost particles are problematic in the analysis, longer minimum trajectory frame-lengths can be used to exclude most of the ghost particles. In summary, it is worthwhile to put effort into the preparation of an imaging experiment. Only then, can one obtain the best result—by applying iterative reconstruction algorithms.

## Figures and Tables

**Table 1:** Typical particle imaging deficits and their influence on the reconstruction algorithms. “Applicable” indicates that the algorithm can be used without major problems.

Given Effect	Shake-The-Box	Triangulation
defocused	problems with ring-shaped particles	applicable after smoothing
particle size <2px	applicable after smoothing	applicable, but inaccurate
intensity saturated	applicable, occlusion not handled	applicable, but inaccurate
low intensity	applicable	applicable
intensity fluctuations	fragmented trajectories	applicable
noisy background	applicable	applicable
